# Introducing mGems, *mBio*’s new review type

**DOI:** 10.1128/mbio.02622-24

**Published:** 2025-01-14

**Authors:** Marcio L. Rodrigues, Jacob S. Yount, Vinayaka R. Prasad, Arturo Casadevall

**Affiliations:** 1 Instituto Carlos Chagas, Fundação Oswaldo Cruz (Fiocruz) 169688 , Curitiba, Brazil; 2 Instituto de Microbiologia Paulo de Góes, Universidade Federal do Rio de Janeiro 2647 , Rio de Janeiro, Brazil; 3 Department of Microbial Infection and Immunity, The Ohio State University, Columbus, Ohio, USA; 4 Infectious Diseases Institute, The Ohio State University 25802 , Columbus, Ohio, USA; 5 Department of Microbiology and Immunology, Albert Einstein College of Medicine, Bronx, New York, USA; 6 Department of Molecular Microbiology and Immunology, Johns Hopkins School of Public Health, Baltimore, Maryland, USA

**Keywords:** mGems, reviews, editorial

## EDITORIAL

The pace of research publishing is continuously accelerating due to the constant expansion of research activities across the world (1). In this context, review articles are valuable tools for the dissemination of science, as they can provide a crash course for new entrants into a field and can help experts stay current (2). For authors, the process of writing a review enhances their expertise, analytical abilities, and communication skills. For readers, a well-crafted review not only synthesizes and interprets the results of a field but also identifies gaps and challenges that future research should address. Integrating review writing into a scientific career can thus have clear benefits (3).

Review articles can impact readers and the areas in which they conduct their science in multiple ways. It is reasonable to expect that experts in a particular field will benefit from highly specialized review articles, whereas newcomers to a specific area will gain more from general overviews and future perspectives. In this case, the content should be easily understandable for those new to the field, which will influence how review articles are written. Experts often overlook the fact that nonspecialists might not draw the same connections or reach the same conclusions. Therefore, it is always necessary to explain all technical terms, clearly articulate the connections between different studies, and provide clear explanations or hypotheses. Use of a language that is easy to understand by individuals representing multiple inter-connected fields is critical to the successful dissemination of the concepts covered in a review. Language that consists of jargon or technical terms that are only popular within a specialized sub-field poses a strong barrier to understanding by all. While concise language should be used, lengthy or convoluted sentences should be avoided (3).

Aligned with this view, *mBio* regularly publishes Minireviews defined as “summaries (with a maximum of 6,000 words and up to two figures or tables) of important developments in microbiology research.” However, the traditional Minireview format, although effective, may not always meet the needs of the rapidly evolving scientific landscape. As the amount of published research continues to grow exponentially, it becomes increasingly challenging to provide comprehensive, yet concise, overviews within the constraints of a Minireview.

To address this gap, we are excited to introduce the mGem format, a shorter, more focused review article that allows for a targeted exploration of specific, cutting-edge topics in microbiology. Inspired by pioneering successful initiatives elsewhere (4), the mGem format (1,500 word limit with up to two figures/tables) is designed to provide quick, yet in-depth, insights into novel research areas, methodologies, or controversies that might not be ideal for a longer review. This new format will serve as a complement to our existing Minireviews, ensuring that both broad and narrowly focused discussions can thrive at *mBio*. Although commissioned contributions will be part of how mGems are published, we encourage authors to submit on any topic related to microbiology. mGems will be collated into a collection that will be featured on the *mBio* collections page (https://journals.asm.org/journal/mbio/collections).

With the launch of mGems, we aim to contribute to the dissemination of knowledge in microbiology and associated areas through dynamic, scientifically and ethically rigorous communication. Start writing your mGem contribution now. We look forward to hearing your views on the microworld!
